# Detection and epidemic dynamic of ToCV and CCYV with *Bemisia tabaci* and weed in Hainan of China

**DOI:** 10.1186/s12985-017-0833-2

**Published:** 2017-09-04

**Authors:** Xin Tang, Xiaobin Shi, Deyong Zhang, Fan Li, Fei Yan, Youjun Zhang, Yong Liu, Xuguo Zhou

**Affiliations:** 1 College of Plant Protection, Hunan Agricultural University, Changsha, 410125 China; 2 Hunan Academy of Agricultural Science, Hunan Plant Protection Institute, Key Laboratory of Pest Management of Horticultural Crop of Hunan Province, No. 726, Yuanda Road, Furong District, Hunan province, Changsha, 410125 China; 3 College of Plant Protection, Yunnan Agricultural University, Yunnan, 650201 China; 4 Institute of virus and biotechnology, Zhejiang Academy of Agricultural Sciences, Hangzhou, 310021 China; 5 Institute of Vegetables and Flowers, Chinese Academy of Agricultural Sciences, Beijing, 100081 China; 6 Department of Entomology, University of Kentucky, S-225 Agricultural Science Center North Lexington, Lexington, KY 40546-0091 USA

**Keywords:** *Tomato chlorosis virus; cucurbit chlorotic yellows virus; Bemisia tabaci*, Molecular identification, Q biotype, *Alternanthera philoxeroides*

## Abstract

**Background:**

In recent years, two of the crinivirus, *Tomato chlorosis virus* (ToCV) and *Cucurbit chlorotic yellows virus* (CCYV) have gained increasing attention due to their rapid spread and devastating impacts on vegetable production worldwide. Both of these viruses are transmitted by the sweet potato whitefly, *Bemisia tabaci* (Gennadius), in a semi-persistent manner. Up to now, there is still lack of report in Hainan, the south of China.

**Methods:**

We used observational and experimental methods to explore the prevalence and incidence dynamic of CCYV and ToCV transmitted by whiteflies in Hainan of China.

**Results:**

In 2016, the chlorosis symptom was observed in the tomato and cucumber plants with a large number of *B. tabaci* on the infected leaves in Hainan, China, with the incidence rate of 69.8% and 62.6% on tomato and cucumber, respectively. Based on molecular identification, Q biotype was determined with a viruliferous rate of 65.0% and 55.0% on the tomato and cucumber plants, respectively. The weed, *Alternanthera philoxeroides* near the tomato and cucumber was co-infected by the two viruses. Furthermore, incidence dynamic of ToCV and CCYV showed a close relationship with the weed, *Alternanthera philoxeroides*, which is widely distributed in Hainan*.*

**Conclusion:**

Our results firstly reveal that the weed, *A. philoxeroides* is infected by both ToCV and CCYV. Besides, whiteflies showed a high viruliferous rate of ToCV and CCYV. Hainan is an extremely important vegetable production and seed breeding center in China. If the whitefly can carry these two viruses concurrently, co-infection in their mutual host plants can lead to devastating losses in the near future.

## Background

Plant virus causes serious threat in the growth and product of crops and vegetables in the world [1]. Plant viruses depend on insect vectors for transmission in a non-persistent, semi-persistent and persistent manner, respectively [2]. The prevalence of plant viruses is closely related to the dynamics of insect vectors [3, 4].

The whitefly, *Bemisia tabaci* (Gennadius) (*hemiptera*; *Aleyrodidae*) is a main vector for plant virus transmission in greenhouse, which has rapidly increased all over the world followed by outbreaks of whitefly-transmitted viruses, causing great losses in agricultural production [5–7]. The most destructive vector in China is *B. tabaci* B (MEAM1) and Q (MED) [8]. *B. tabaci* B has been documented in China since the mid-1990’s, but *Tomato yellow leaf curl virus* (TYLCV) was not detected until Q became established in 2003 [9, 10], and epidemic of TYLCV is associated with the increasing number of Q [10, 11]. Plant virus can be transmitted by whiteflies in a persistent or semi-persistent manner. Up to now, most research has been focused on the persistent-transmitted virus such as TYLCV but less attention is paid on semi-persistent transmitted viruses. To note, research on the relationship between epidemiology of the crinivirus and whitefly is important to prevent virus outbreak.

*Tomato chlorosis virus* (ToCV), genus crinivirus, family *closteroviridae* [12], is transmitted by *B. tabaci* in a semi-persistent manner [1, 13]. The disorder and yellow symptoms such as the interveinal chlorosis, the leaf brittleness, and the limited necrotic flecking can be used to determine the virus [1, 14, 15]. ToCV was first reported in Florida [16], and then it transmitted to Spain [17], Africa [18, 19], the Middle East [17], and Asia [20, 21]. ToCV can be infected in 24 species of 7 family plants [1]. In Spain Q whiteflies has been determined on ToCV-infected leaves [17]. In Costa Rica Q whiteflies has also been detected on ToCV-infected leaves [22]. In China ToCV was first found in Taiwan [23], and then was found in Shandong [21] and many other northern places, such as Shanxi, Beijing and Neimenggu [24]. Up to now, there is still lack of report in the south of mainland China such as Hainan. With the increasing number of whiteflies in recent years, the potential threat should be noticed.

*Cucurbit chlorotic yellows virus* (CCYV) belongs to genus crinivirus, family *closteroviridae* [25]. CCYV can cause chlorotic leaf spots and yellowing of leaves in pumpkin, melon, watermelon, and tobacco [25, 26]. CCYV is transmitted by *B. tabaci* B and Q in a semi-persistent manner. It was first determined in Japan in 2010 [27], and then it was found in China [28], Sudan [29], Greece [30] and Iran [31]. CCYV was also found in many northern places in China, such as Beijing, Hebei, and Anhui provinces (unpublished data). There is still lack of reports on whitefly biotype detection on virus-infected plants, which has an important role in research of the relationship between epidemiology of the crinivirus and whitefly.

In this research, we found the severe typical chlorotic symptoms on tomato and cucumber in many vegetable growing areas in Hainan province—the south of China. We found that numerous whiteflies gathered on infected plant leaves in cultivated places. We then collected the whiteflies and infected leaves with typical symptoms and then brought them into laboratory to detect the whitefly biotype and to determine the virus. ToCV and CCYV were identified, and *B. tabaci* Q was determined in all infected leaves. The weeds, *Alternanthera philoxeroides* nearby were also collected and determined, and the dynamics of ToCV and CCYV were then determined on tomato, cucumber and weeds in four growth stages in Yongfazhen where ToCV and CCYV showed a high virus incidence. Our results provide a basis for monitoring and prevention of viral diseases.

## Methods

### Field survey

To determine the incidence of the chlorosis disease in tomato and cucumber crops, a survey was undertaken in the Hainan province. Five sites (Yunlongzhen, Xinzhuzhen, Yongfazhen, Tianyazhen, and Yachengzhen), representing the main vegetable-growing areas were surveyed (Fi﻿g﻿. 1). For each site, over 200 to 300 plants including tomato and cucumber were surveyed and the incidence of the chlorosis disease was calculated. At each site, the chlorosis tomato and cucumber plants were selected and taken to the laboratory for molecular detection. Meanwhile, whiteflies on the symptomatic plants were collect randomly with aspirating equipment and taken to the laboratory for molecular detection.

**Fig. 1:**
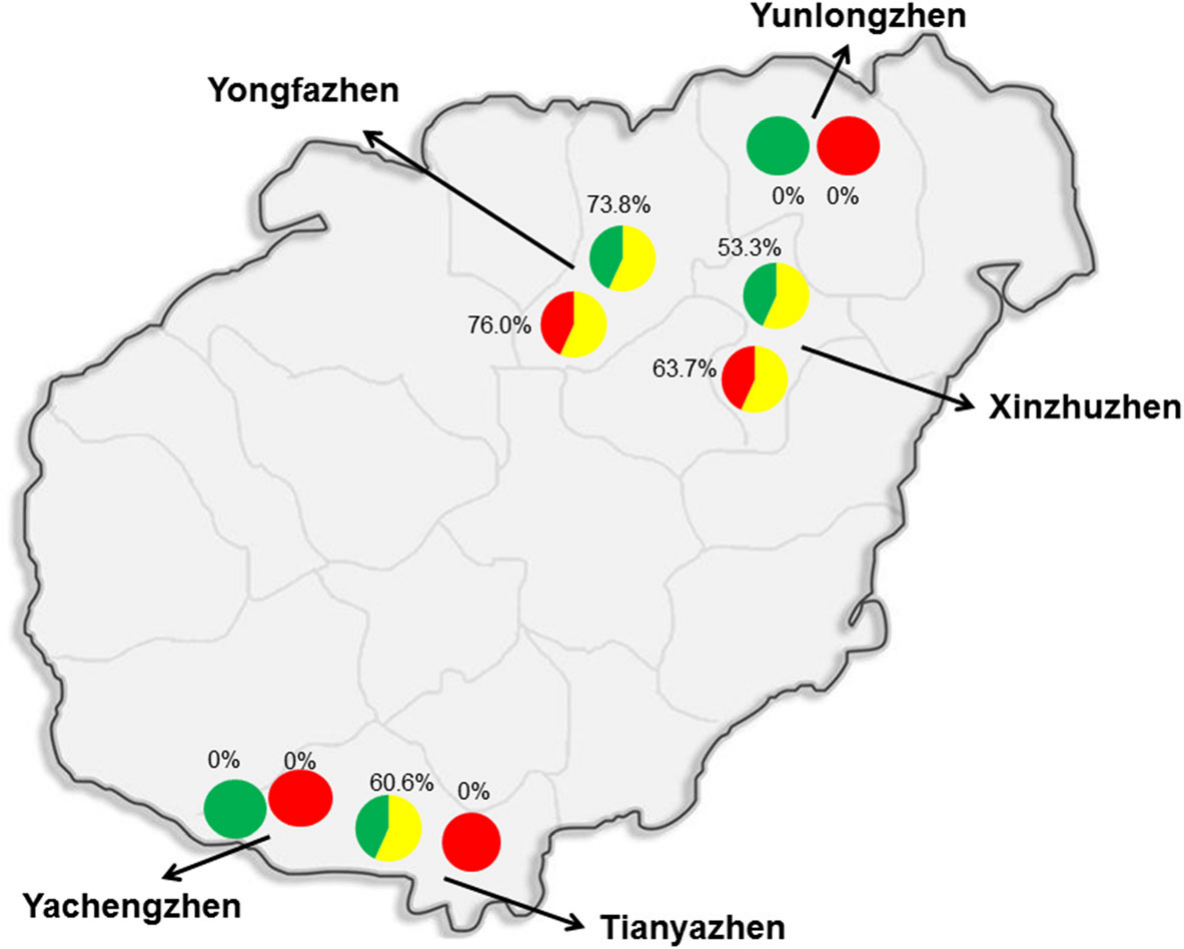
Geographic locations of surveyed plants. Five sites in Yunlongzhen, Yongfazhen, Xinzhuzhen, Yachengzhen, and Tianyazhen were shown in the figure. In each site tomato and cucumber plants were investigated and sampled. Red circles and sectors represent tomato plants. Green circles and sectors represent cucumber plants. Yellow sectors represent virus-infected tomato and cucumber plants

### Whitefly biotype and viruliferous rate detection

The whitefly samples were divided into four parts, of which two parts were used to detect the whitefly biotype on tomato and cucumber, and the other two parts were used to detect the viruliferous rate of ToCV and CCYV. The whitefly biotype was detected using the CAPS-cleavage amplified polymorphic sequence of *mitochondrial cytochrome oxidase I* gene (*mtCOI*) with the restriction endonuclease *AseI* [32]. The viruliferous rate detection method was described in section of virus detection in plants. In each part, 20 whiteflies were detected, and each of the detection was repeated three times.

### RNA extraction and reverse transcription from infected leaves

Total RNA was extracted separately from 0.1 g infected tomato and cucumber leaves using the total RNA extraction kit (Tiangen Biotech, Beijing, China) following the manufacturer’s instruction. Each of 20 samples was extracted from tomato and cucumber respectively. Each of the detection was repeated three times. Reverse transcription of RNA from the total nucleic acid extracts was performed using cDNA synthesis kit (Takara, Beijing, China), following the manufacturer’s instruction.

### Virus detection in plants

ToCV detection: We selected 60 chlorosis tomato leaves for detection, of which 20 leaves were detected each time, and all the leaves were detected for 3 times. Reverse transcript–polymerase chain reaction (RT-PCR) was carried out using the primers designed in the HSP70h gene of ToCV using Primer Premier 5 software (Table 1). The PCR of ToCV was performed in 20 μl of reaction mixtures containing 7 μl of ddH_2_O, 10 μl of mix, 1 μl of each primer, and 1 μl of cDNA. The PCR procedures are as follows: initial denaturation at 94 °C for 2 min, followed by 35 cycles of 94 °C for 15 s, 56 °C for 30 s and 72 °C for 30s, and a final elongation step at 72 °C for 10 min. The PCR products of ToCV were obtained and then separated by electrophoresis using 1.0% agarose gels.

**Table 1:** Primers of the ToCV, CCYV and whitefly

Name	Primer	Sequence
ToCV	F	AAACTGCCTGCATGAAAAGTCTC
	R	GGTTTGGATTTTGGTACTACATTCAGT
CCYV	F	CGCAATCAATAAGGCGGCGACC
	R	ACTACAACCTCCCGGTGCCAACT
Whitefly	F	TTGATTTTTTGGTCATCCAGAAGT
	R	CTGAATATCGRCGAGGCATTCC

CCYV detection: We selected 60 chlorosis cucumber leaves for detection, of which 20 leaves were detected each time, and all the cucumber leaves were detected for 3 times. The PCR of the cucumber samples was carried out using the primers designed in the coat protein (CP) gene of CCYV using Primer Premier 5 software (Table 1). The PCR of CCYV was performed in 20 μl of reaction mixtures including 7 μl of ddH_2_O, 10 μl of mix, 1 μl of each primer, and 1 μl of cDNA. The PCR procedures are as follows: initial denaturation at 94 °C for 5 min, followed by 35 cycles of 94 °C for 15 s, 53 °C for 30 s and 72 °C for 1 min, and a final extension at 72 °C for 10 min. The PCR products of CCYV were obtained and then separated by electrophoresis using 1.0% agarose gels.

ToCV and CCYV detection on weeds: In Yongfazhen, where ToCV and CCYV were detected with a high incidence, we collected the weeds, *A. philoxeroides* which are close to the infected tomato and cucumber to detect ToCV and CCYV. We collected 60 weed leaves for detection of ToCV and CCYV in three replicates.

### Nucleotide sequencing analysis

The target PCR products were purified by the AxyPrep DNA gel extraction kit (Axygen, Zhejiang, China), following the manufacturer’s instructions. The purified products were then sequenced at the Sangon biotech (Shanghai, China). The sequence data of the whiteflies, ToCV and CCYV on tomato, cucumber and weeds were analysed using the BioEdit software. Sequences were compared with the NCBI nucleotide database via the BLAST tools on NCBI online server.

### Virus incidence dynamic on tomato, cucumber and weeds

The incidence dynamics of ToCV and CCYV were determined on tomato, cucumber and the weeds nearby: In four growth stages, transplanting, seedling, flowering and ripening of tomato and cucumber, plants were collected to our lab to detect the viruliferous rate of ToCV and CCYV. The weed, *A. philoxeroides* that was grown near the tomato and cucumber plants was also collected to detect the viruliferous rate of ToCV and CCYV. In each of the five sites of Yongfazhen where ToCV and CCYV were detected with a high incidence, 100 tomato leaves were collected for detection of ToCV, and 100 cucumber leaves were collected for detection of CCYV. The tomato plants and cucumber plants were adjacent, therefore 100 weed leaves nearby were collected for detection of ToCV and CCYV. That is to say, 500 tomato leaves, 500 cucumber leaves and 500 weed leaves were collected in one growth stage.

### Data analysis

Statistical analyses were performed with SPSS (version 19.0, Chicago, IL, USA). One-way ANOVA was used to compare the viruliferous rate of plants in different growth stages and weeds.

## Results

### Incidence of chlorosis disease

The total number of 300 tomato plants and 240 cucumber plants was count in infected places of Xinzhuzhen, Yongfazhen and Yazhouzhen to calculate the virus incidence. The total number of 210 tomato plants and 150 cucumber plants was observed to show chlorosis symptom, with the average incidence of 69.8% and 62.6%, respectively (Fig. 1, Table 2). In Yongfazhen, the viruliferous rate of ToCV and CCYV on the weed was 15.0% and 11.7%, respectively (Fig﻿﻿. 1, Table 2).

**Table 2:** Incidence of ToCV and CCYV

Virus	Host plants	Geographic locations	Number of plants surveyed	Number of infected plants	Incidence of chlorosis disease (%)	Average incidence of chlorosis disease (%)
ToCV	Tomato	Yunlongzhen	145	0	0.0	69.8
		Xinzhuzhen	146	93	63.7	
		Yongfazhen	154	117	76.0	
		Yachengzhen	165	0	0.0	
		Tianyazhen	150	0	0.0	
CCYV	Cucumber	Yunlongzhen	92	0	0.0	62.6
		Xinzhuzhen	90	48	53.3	
		Yongfazhen	84	62	73.8	
		Yachengzhen	66	40	60.6	
		Tianyazhen	81	0	0.0	
ToCV	Weed	Yongfazhen	60	9	15.0	15.0
CCYV	Weed	Yongfazhen	60	7	11.7	11.7

### Whitefly biotype and viruliferous rate detection

**Fig. 2:**
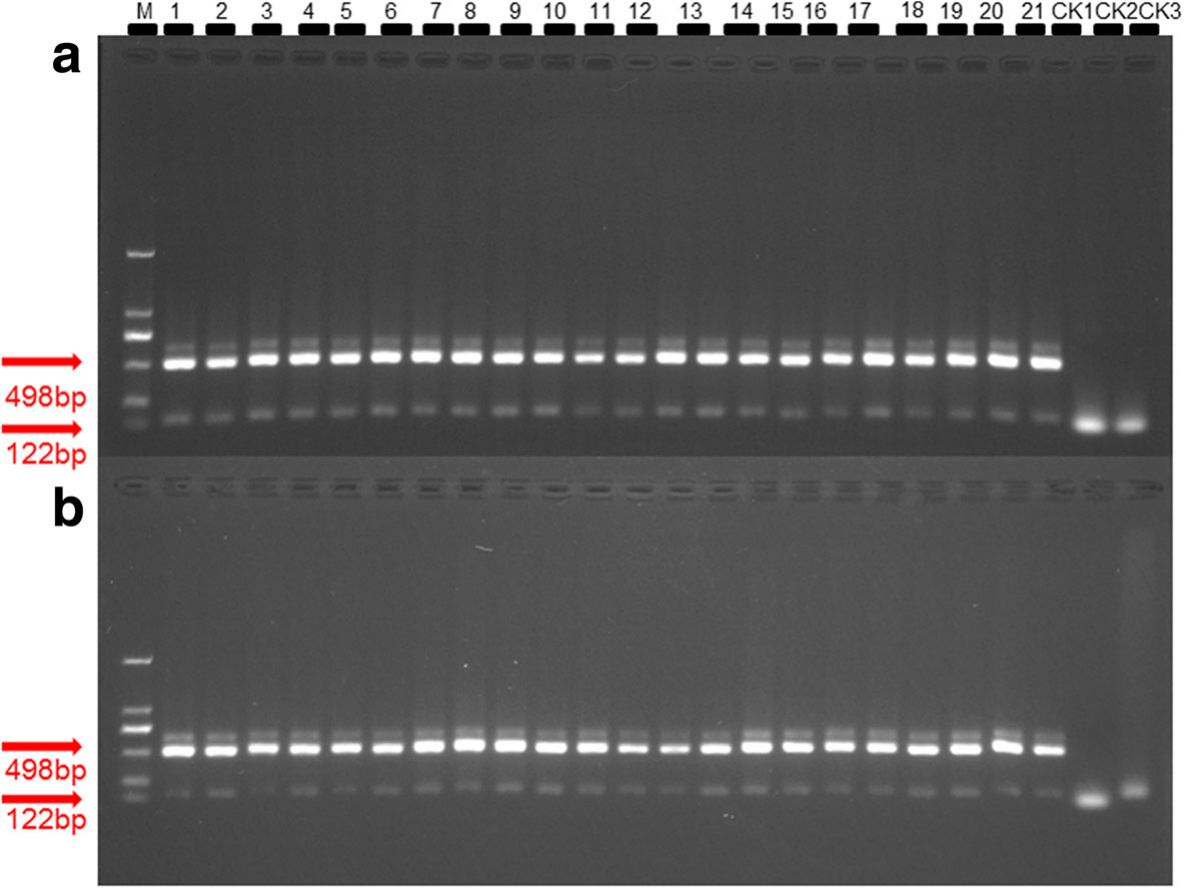
Whitefly biotype detection. CK1 positive control; CK2 negative control; CK3 black control. **a** Whitefly biotype on infected tomato plants. **b** Whitefly biotype on infected cucumber plants. The size of 122 bp and 498 bp fragment based on amplification of *mtCOI* and restriction endonuclease was used to detect the biotype. Results of 20 samples were shown in figure **a** and **b**

PCR amplification confirmed that all the whiteflies gathered in symptomatic tomato and cucumber plants were *B. tabaci* Q. The percentage of viruliferous whitefly was 65.0% and 55.0%, respectively (Fig. 2; Table 3).

**Table 3:** Whitefly biotype and viruliferous rate

Virus	Number of whiteflies	Whitefly biotype	Number of viruliferous whiteflies	Viruliferous rate
ToCV	60	Q	39	65.0%
CCYV	60	Q	33	55.0%

### Virus detection in plants

**Fig. 3:**
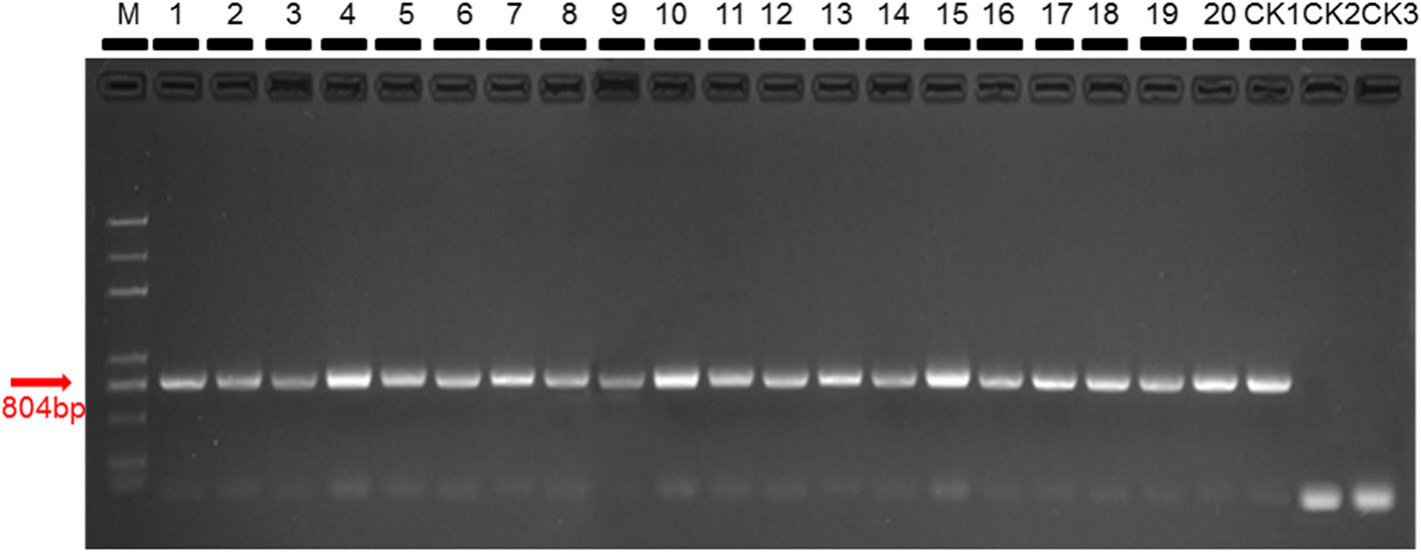
ToCV detection from tomato plants. CK1 positive control; CK2 negative control; CK3 black control. The size of 466 bp based on amplification of HSP70h gene of ToCV was used. Results of 20 samples were shown in this figure

The size of 804 bp based on amplification of CP (coat protein) gene of CCYV was amplified, which revealed that the symptomatic cucumber plants collected in Xinzhuzhen, Yongfazhen and Yazhouzhen and the weeds collected in Yongfazhen of Hainan province was infected by CCYV (Figs. 3 and 5a).

**Fig. 4:**
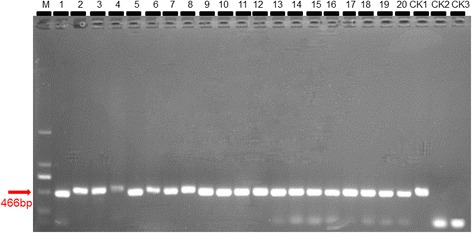
CCYV detection from cucumber plants. CK1 positive control; CK2 negative control; CK3 black control. The size of 804 bp based on amplification of CP (coat protein) gene of CCYV was used. Results of 20 samples were shown in this figure

**Fig. 5:**
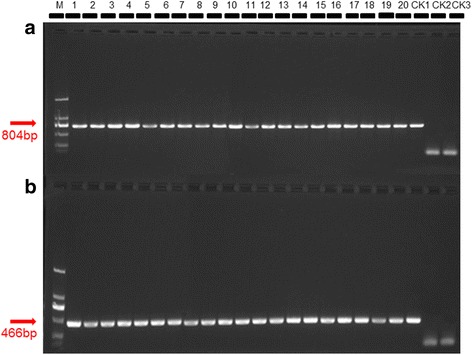
ToCV and CCYV detection from weeds. **a** CCYV detection on weeds. **b** ToCV detection on weeds. CK1 positive control; CK2 negative control; CK3 black control. The size of 804 bp based on amplification of CP (coat protein) gene of CCYV and the size of 466 bp based on amplification of HSP70h gene of ToCV were used. Results of 20 samples were shown in figure **a** and **b**

The size of 466 bp based on amplification of heat shock 70-like protein (HSP70h) gene of ToCV was amplified, which showed that the chlorosis tomato plants collected in Xinzhuzhen and Yongfazhen and the weeds collected in Yongfazhen of Hainan province was infected by ToCV (Figs. 4 and 5b).

### Nucleotide sequencing analysis

The sequencing results were shown in Table 4. The sequence of the whitefly samples shows a similarity of 99% with the *cytochrome oxidase subunit I (COI)* gene of *B. tabaci* Q (KT265875.1). Virus samples in tomato were 100% similar with the RNA1 of ToCV (KC887999.1), and the virus samples in cucumber showed 97% similar with the CP gene of CCYV (KX118632.1). The virus samples in weed showed a similarity of 99% and 97% with the RNA1 of ToCV (KC887999.1) and the CP of CCYV (KX118632.1), respectively.

**Table 4:** Nucleotide sequencing analysis of *B. tabaci* and plant viruses

Sample	Sequencing description	Accession	Max score	Total score	Query cover	E value	Identities
Tomato	*Tomato chlorosis virus* isolate ToCV-BJ segment RNA2	KC887999.1	863	863	45%	0.0	100%
Cucumber	*Cucurbit chlorotic yellows virus* isolate GX-BH capsid protein gene	KX118632.1	1354	1354	97%	0.0	99%
*B. tabaci*	*Bemisia tabaci* biotype Q cytochrome oxidase subunit 1 (COI) gene	KT265875.1	1062	1062	99%	0.0	99%
Weed	*Tomato chlorosis virus* isolate ToCV-BJ segment RNA2	KC887999.1	856	856	41%	0.0	99%
Weed	*Cucurbit chlorotic yellows virus* isolate GX-BH capsid protein gene	KX118632.1	1055	1055	77%	0.0	97%

### Virus incidence dynamic on tomato, cucumber and weeds

**Fig. 6:**
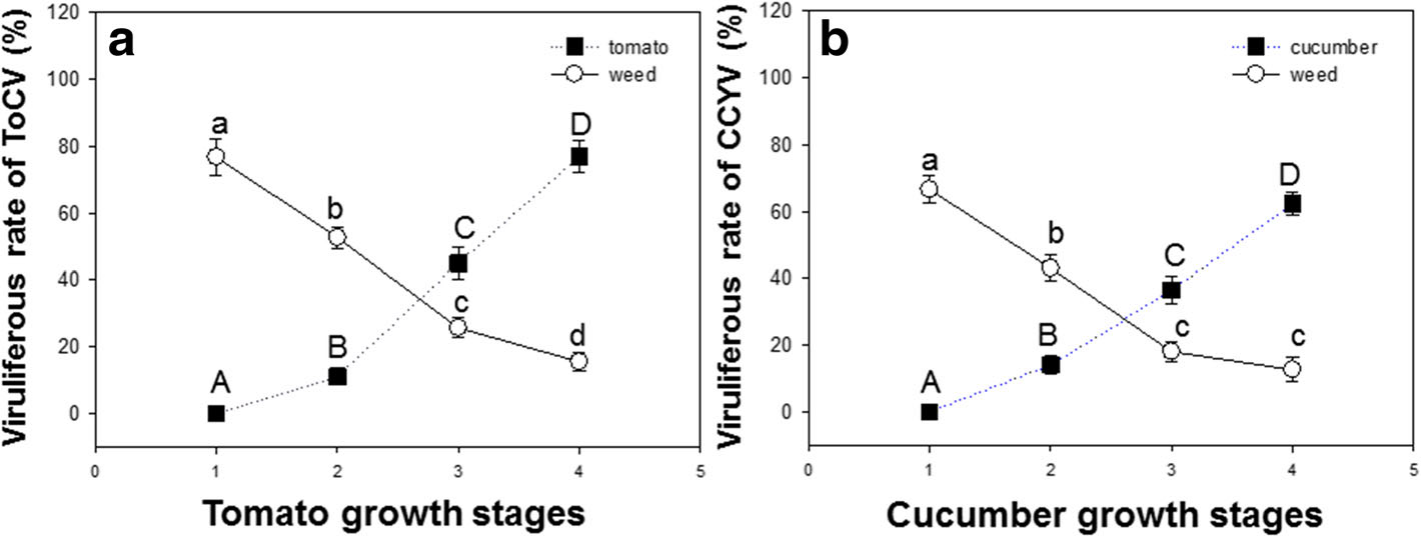
Virus incidence dynamic on tomato, cucumber and weeds in four of plant growth stages. **a** Viruliferous rate of ToCV in four of the tomato growth stages. **b** Viruliferous rate of CCYV in four of the cucumber growth stages. Values are means ± SE. 1 transplanting stage; 2 seedling stage; 3 flowering stage; 4 ripening stage. On each growth stage, different lowercase letters of a-d indicate significant differences on weed and different uppercase letters of A-D indicate significant differences on plants (*P* < 0.05)

Virus incidence dynamic of ToCV on tomato, and the weed, *A. philoxeroides* and CCYV on cucumber and *A. philoxeroides* changes significantly in the four growth stages of plants (ToCV on tomato: *F*_3,16_ = 160.737, *P* < 0.001; ToCV on weed: *F*_3,16_ = 91.701, *P* < 0.001; CCYV on cucumber: *F*_3,16_ = 136.496, *P* < 0.001; CCYV on weed: *F*_3,16_ = 75.522, *P* < 0.001). In the transplanting stage, viruliferous rate of both ToCV on tomato and CCYV on cucumber was 0%. However, in the ripening stage, viruliferous rate of ToCV and CCYV on tomato and cucumber was highest, with the viruliferous rate of 77% and 62.4%, respectively. Viruliferous rate of ToCV and CCYV on *A. philoxeroides* showed an opposite trend, which was highest in the transplanting stage of plants and lowest in the ripening stage of plants. In the transplanting stage, the viruliferous rate of ToCV and CCYV on *A. philoxeroides* was 76.8% and 66.6%, respectively. In the ripening stage, the viruliferous rate of ToCV and CCYV on *A. philoxeroides* was 15.4% and 12.6%, respectively. Notably, the weed *A. philoxeroides* that was adjacent from tomato and cucumber can carry both ToCV and CCYV at the same time, with the co-infection viruliferous rate of 32.2% and 6.4% in the transplanting stage and ripening stage, respectively (Fig. 6).

## Discussion

*B. tabaci* is a most important insect vector in agricultural areas and has caused great losses in economy and crop production worldwide [25, 33]. The indirect damage caused by virus transmission is much serious than the direct feeding on the host. For example, TYLCV is transmitted by whitefly in a persistent manner, which causes destructive damage in China [9, 34]. In recent years, TYLCV has attracted the large attention and a series of measure has been used to prevent the disease by researchers in China [11, 35–37]. However, up to now, we still pay less attention to most of the semi-persistent viruses transmitted by the whitefly. Furthermore, those plant viruses such as ToCV and CCYV are huge potential crises to agricultural production.

In our research, we found that high density rates of Q at open field in Hainan province and at the same areas we determined that the leaves were infected by ToCV and CCYV, with the incidence of 69.8% and 62.6% on tomato and cucumber plants, respectively. Besides, the viruliferous rate of Q was 65.0% and 55.0% on the tomato and cucumber plants, respectively. Plant virus disease prevalence is closely related to the spread of insect vector. Although *B. tabaci* B has been shown to be an effective vector of ToCV [1], recently, Q has become a major threat to the quality and yields by transmitting ToCV [22]. Besides, *B. tabaci* Q plays more roles than B in carrying CCYV [38]. In this research, we notice that the prevalence of CCYV in cucumber and ToCV in tomato was high which was consistent with the high viruliferous rate of Q. Therefore we can speculate that high viruliferous rate of Q may facilitate transmission of ToCV and CCYV.

The weed *A. philoxeroides*, which was grown near the infected tomato and cucumber, was also infected by ToCV and CCYV. The virus dynamic was then detected on tomato, cucumber and the weeds nearby. In the four growth stages, virus showed a different dynamic on plants and on weeds. On tomato and cucumber plants, viruliferous rate of ToCV and CCYV increased gradually from transplanting stage to ripening stage. On weeds, viruliferous rate of ToCV and CCYV decreased gradually from ripening stage to transplanting stage. Furthermore, both of the ToCV and CCYV were detected on the weed, *A. philoxeroides*, which is a widely distributed weed in Hainan. To our knowledge, this is the first report of ToCV and CCYV on the weed, *A. philoxeroides*. From our results we can see that the weed, *A. philoxeroides* is co-infected and may promote the virus transmission, it’s a pity that we didn’t detect whether the whiteflies were co-infected on weeds, and this needs further confirmation. Notably, Hainan is the mainly vegetable production and breeding center especially for breeding tomato and cucumber in China. In winter season, vegetables in Hainan are transported to all of the north provinces of China because of the low temperature in north provinces. Therefore, the break out of these two viruses may cause fast transmission of ToCV and CCYV to other places via infected seed or viruliferous whiteflies.

## Conclusion

This report firstly shows ToCV and CCYV detected in the same area with a high incidence in Hainan province, with a high viruliferous rate of Q on infected leaves. Furthermore, the virus dynamic shows a close relationship with the weed nearby, and the weed is infected by both ToCV and CCYV. Hainan is an extremely important vegetable production and seed breeding center in China. If the whitefly can carry these two viruses concurrently, co-infection in their mutual host plants can lead to devastating losses in the near future. Further research should be done to investigate the role of weed in the transmission of virus.

## Abbreviations

CCYV: *Cucurbit chlorotic yellows virus*
CP: Coat protein
HSP70h: Heat shock 70-like protein
RT-PCR: Reverse transcript–polymerase chain reaction
ToCV: *Tomato chlorosis virus*
TYLCV: *Tomato yellow leaf curl virus*

## References

[1] Wintermantel WM, Wisler GC (2006). Vector specificity, host range, and genetic diversity of *Tomato chlorosis virus*. Plant Dis.

[2] Brault V, Uzest M, Monsion B, Jacquot E, Blanc S (2010). Aphids as transport devices for plant viruses. C R Biol.

[3] Pinheiro PV, Kliot A, Ghanim M, Cilia M (2015). Is there a role for symbiotic bacteria in plant virus transmission by insects?. Curr Opin Insect Sci..

[4] Whitfield AE, Rotenberg D (2015). Disruption of insect transmission of plant viruses. Curr Opin Insect Sci.

[5] Wintermantel WM. Transmission efficiency and epidemiology of criniviruses. In *Bemisia*: bionomics and management of a global pest*.* Springer. 2009; 319–331.

[6] Navas-Castillo J, Fiallo-Olivé E, Sánchez-Campos S (2011). Emerging virus diseases transmitted by whiteflies. Annu Rev Phytopathol.

[7] Wisler G, Duffus J, Liu HY, Li R (1998). Ecology and epidemiology of whitefly-transmitted closteroviruses. Plant Dis.

[8] De Barro PJ, Liu SS, Boykin LM, Dinsdale AB (2011). *Bemisia tabaci*: a statement of species status. Annu Rev Entomol.

[9] Pan H, Chu D, Yan W, Su Q, Liu B, Wang S, Wu Q, Xie W, Jiao X, Li R (2012). Rapid spread of *Tomato yellow leaf curl virus* in China is aided differentially by two invasive whiteflies. PLoS One.

[10] Pan H, Chu D, Liu B, Shi X, Guo L, Xie W, Carriere Y, Li X, Zhang Y (2013). Differential effects of an exotic plant virus on its two closely related vectors. Sci Rep-UK..

[11] Shi X, Pan H, Zhang H, Jiao X, Xie W, Wu Q, Wang S, Fang Y, Chen G, Zhou X (2014). *Bemisia tabaci* Q carrying *Tomato yellow leaf curl virus* strongly suppresses host plant defenses. Sci Rep-UK..

[12] Wintermantel WM, Wisler GC, Anchieta AG, Liu HY, Karasev AV, Tzanetakis IE (2005). The complete nucleotide sequence and genome organization of *Tomato chlorosis virus*. Arch Virol.

[13] Orfanidou C, Pappi P, Efthimiou K, Katis N, Maliogka V (2016). Transmission of *Tomato chlorosis virus* (ToCV) by *Bemisia tabaci* biotype Q and evaluation of four weed species as viral sources. Plant Dis.

[14] Fortes IM, Navas-Castillo J (2012). Potato, an experimental and natural host of the crinivirus *Tomato chlorosis virus*. Eur J Plant Pathol.

[15] García-Cano E, Navas-Castillo J, Moriones E, Fernández-Muñoz R (2010). Resistance to *Tomato chlorosis virus* in wild tomato species that impair virus accumulation and disease symptom expression. Phytopathology.

[16] Wisler GC, Li RH, Liu HY, Lowry DS, Duffus JE (1998). *Tomato chlorosis virus*: a new whitefly-transmitted, phloem-limited, bipartite closterovirus of tomato. Phytopathology.

[17] Navas-Castillo J, Camero R, Bueno M, Moriones E (2000). Severe yellowing outbreaks in tomato in Spain associated with infections of *Tomato chlorosis virus*. Plant Dis.

[18] Gharsallah C, Halima AB, Fakhfakh H, Gorsane F (2014). Insights into the genetic diversity and the phylogenetic analysis of Tunisian isolates of *Tomato chlorosis virus*. Phytoparasitica.

[19] Moodley V, Gubba A, Mafongoya P. Occurrence of *Tomato chlorosis virus* (ToCV) on *Datura stramonium* near tomato crops (*Solanum lycopersicum*) in South Africa. Plant Dis. 2016;

[20] Kil EJ, Lee YJ, Cho S, Auh CK, Kim D, Lee KY, Kim MK, Choi HS, Kim CS, Lee S (2015). Identification of natural weed hosts of *Tomato chlorosis virus* in Korea by RT-PCR with root tissues. Eur J Plant Pathol.

[21] Zhao LM, Li G, Gao Y, Liu YJ, Sun GZ, Zhu XP (2014). Molecular detection and complete genome sequences of *Tomato chlorosis virus* isolates from infectious outbreaks in China. J Phytopathol.

[22] Guevara-Coto JA, Barboza-Vargas N, Hernandez-Jimenez E, Hammond RW, Ramirez-Fonseca P (2011). *Bemisia tabaci* biotype Q is present in Costa Rica. Eur J Plant Pathol.

[23] Tsai W, Shih S, Green S, Hanson P, Liu H. First report of the occurrence of *Tomato chlorosis virus* and *Tomato infectious chlorosis virus* in Taiwan. Plant Dis 2004; 88:311–311. 10.1094/PDIS.2004.88.3.311B 30812372

[24] Zheng HX, Xia JX, Zhou XM, Zhang YJ. Be on alert of rapid diffusion of *Toamto chlorosis virus* transmitted by whitefly in China. China Vegetables. 2016; 22–26 (Chinese with English abstrct).

[25] Abrahamian PE, Abou-Jawdah Y (2014). Whitefly-transmitted criniviruses of cucurbits: current status and future prospects. Virus disease.

[26] Orfanidou C, Maliogka V, Katis N. First report of *Cucurbit chlorotic yellows virus* in cucumber, melon, and watermelon in Greece. Plant Dis 2015; 99:734–734. 10.1094/PDIS-03-14-0311-PDN 30703984

[27] Okuda M, Okazaki S, Yamasaki S, Okuda S, Sugiyama M (2010). Host range and complete genome sequence of *Cucurbit chlorotic yellows virus*, a new member of the genus crinivirus. Phytopathology.

[28] Zeng R, Dai FM, Chen WJ, Lu JP. First report of *Cucurbit chlorotic yellows virus* infecting melon in China. Plant Dis 2011; 95:354–354. 10.1094/PDIS-08-10-0613 30743541

[29] Hamed K, Menzel W, Dafalla G, Gadelseed AMA, Winter S. First report of *Cucurbit chlorotic yellows virus* infecting muskmelon and cucumber in Sudan. Plant Dis 2011; 95:1321–1321. 10.1094/PDIS-04-11-0349 30731657

[30] Orfanidou C, Maliogka VI, Katis NI. First report of *Cucurbit chlorotic yellows virus* in cucumber, melon, and watermelon in Greece. Plant Dis 2014; 98:1446–1446. 10.1094/PDIS-03-14-0311-PDN 30703984

[31] Bananej K, Menzel W, Kianfar N, Vahdat A, Winter S. First report of *Cucurbit chlorotic yellows virus* infecting cucumber, melon, and squash in Iran. Plant Dis 2013; 97:1005–1005. 10.1094/PDIS-01-13-0125-PDN 30722569

[32] Chu D, Wan FH, Zhang YJ, Brown JK (2010). Change in the biotype composition of *Bemisia tabaci* in shandong province of China from 2005 to 2008. Environ Entomol.

[33] Brown JK, Czosnek H (2002). Whitefly transmission of plant viruses. Adv Bot Res.

[34] Liu B, Preisser EL, Chu D, Pan H, Xie W, Wang S, Wu Q, Zhou X, Zhang Y (2013). Multiple forms of vector manipulation by a plant-infecting virus: *Bemisia tabaci* and *Tomato yellow leaf curl virus*. J Virol.

[35] Shi X, Pan H, Xie W, Wu Q, Wang S, Liu Y, Fang Y, Chen G, Gao X, Zhang Y (2013). Plant virus differentially alters the plant's defense response to its closely related vectors. PLoS One.

[36] Fang Y, Jiao X, Xie W, Wang S, Wu Q, Shi X, Chen G, Su Q, Yang X, Pan H (2013). *Tomato yellow leaf curl virus* alters the host preferences of its vector *Bemisia tabaci*. Sci Rep-UK..

[37] Ning W, Shi X, Liu B, Pan H, Wei W, Zeng Y, Sun X, Xie W, Wang S, Wu Q (2015). Transmission of *Tomato yellow leaf curl virus* by *Bemisia tabaci* as affected by whitefly sex and biotype. Sci Rep-UK.

[38] Lu SH (2017). Li JH, Wang XL, Song DY, Bai R, Shi Y, Gu QS, Kuo YW, Falk BW. Yan FM A semipersistent plant virus differentially manipulates feeding behaviors of different sexes and biotypes of its whitefly vector Viruses.

