# Correction to: Detection and epidemic dynamic of ToCV and CCYV with *Bemisia tabaci* and weed in Hainan of China

**DOI:** 10.1186/s12985-019-1121-0

**Published:** 2019-01-30

**Authors:** Xin Tang, Xiaobin Shi, Deyong Zhang, Fan Li, Fei Yan, Youjun Zhang, Yong Liu, Xuguo Zhou

**Affiliations:** 1 College of Plant Protection, Hunan Agricultural University, Changsha, 410125 China; 2 Hunan Academy of Agricultural Science, Hunan Plant Protection Institute, Key Laboratory of Pest Management of Horticultural Crop of Hunan Province, No. 726, Yuanda Road, Furong District, Hunan province, Changsha, 410125 China; 3 College of Plant Protection, Yunnan Agricultural University, Yunnan, 650201 China; 4 Institute of virus and biotechnology, Zhejiang Academy of Agricultural Sciences, Hangzhou, 310021 China; 5 Institute of Vegetables and Flowers, Chinese Academy of Agricultural Sciences, Beijing, 100081 China; 6 Department of Entomology, University of Kentucky, S-225 Agricultural Science Center North Lexington, Lexington, KY 40546-0091 USA

**Correction to: Virol J**

**https://doi.org/10.1186/s12985-017-0833-2**

In the original publication of this article [1], the author found the legends of Fig. 3 and Fig. 4 were incorrect.

The original ones were:

- Fig. 3 ToCV detection from tomato plants. CK1 positive control; CK2 negative control; CK3 black control. The size of 466 bp based on amplification of HSP70h gene of ToCV was used. Results of 20 samples were shown in this figure
- Fig. 4 CCYV detection from cucumber plants. CK1 positive control; CK2 negative control; CK3 black control. The size of 804 bp based on amplification of CP (coat protein) gene of CCYV was used. Results of 20 samples were shown in this figure

The correct ones should be:

- Fig. 3 CCYV detection from cucumber plants. CK1 positive control; CK2 negative control; CK3 black control. The size of 804 bp based on amplification of CP (coat protein) gene of CCYV was used. Results of 20 samples were shown in this figure
- Fig. 4 ToCV detection from tomato plants. CK1 positive control; CK2 negative control; CK3 black control. The size of 466 bp based on amplification of HSP70h gene of ToCV was used. Results of 20 samples were shown in this figure
